# Long non coding RNAs and ALS: Still much to do

**DOI:** 10.1016/j.ncrna.2018.11.004

**Published:** 2018-11-15

**Authors:** Stella Gagliardi, Cecilia Pandini, Maria Garofalo, Matteo Bordoni, Orietta Pansarasa, Cristina Cereda

**Affiliations:** a Genomic and Post-Genomic Center, IRCCS Mondino Foundation, Pavia, Italy; b Department of Biology and Biotechnology “L. Spallanzani”, University of Pavia, Pavia, Italy

**Keywords:** ALS, lncRNAs

## Abstract

Alterations in RNA metabolism play an important role in Amyotrophic Lateral Sclerosis (ALS) pathogenesis. The literature has described, so far, a small number of long non coding RNAs (lncRNAs) associated to ALS demonstrating that how there is still much to do to identify and understand their role in ALS. This class of RNAs may offer numerous starting points for new investigations about pathogenic mechanism involved in ALS disease. In this review, we have collected all the presented data about lncRNAs and ALS to offer an overview about this class of non-coding RNAs and their possible role in ALS disease.

## 1 Amyotrophic lateral sclerosis and RNA regulation

Amyotrophic Lateral Sclerosis (ALS) is a progressive and fatal neurodegenerative disease, caused by the selective loss of both upper and lower motor neurons in the cerebral cortex, brainstem and spinal cord [1]. Approximately 90–95% of cases are classified as sporadic ALS (SALS). The remaining 5–10% of cases are familial (FALS). Both SALS and FALS forms can present mutations in candidate genes; the most common mutated genes are SOD1, TARDBP and FUS characterized by point mutations [2] and also C9orf72 gene defined to the hexanucleotide repeat expansion [3]. ALS is a complex and multifactorial disease with many altered cellular processes but, in the last few years, there is mounting evidence that altered RNA metabolism plays an important role in ALS pathogenesis [[4], [5], [6]]. This concept is supported by the discovery of causative genetic mutations in FUS/TLS and in TARDBP genes coding for DNA/RNA binding proteins (RBPs) involved in transcription and RNA metabolism [7]. The same notion is strengthened by the observation that ANG, where four mutations in the coding region of the gene were identified in 298 ALS patients [8], is involved in processing of RNAs and that C9orf72 repeats forms RNA foci in the nucleus of the cells in particular in neuron, and sequester key RBPs leading to impairment in RNA processing events [9,10].

Hexanucleotide repeat expansion in C9ORF72 produces dipeptide repeat proteins (DPRs), in particular polyGly-Argn(GR) and polyPro-Arg (PR) [11], that disturb phase transitions mediated by low complexity sequence domains (LCDs). The altered assembly, dynamics, and function of membrane-less organelle that result from disturbed phase transitions fully account for the widespread cellular abnormalities observed in ALS [12].

Moreover, important ALS genes, such as SOD1 have been described deregulated at mRNAs level [13,14]. In fact, even if SOD1, TDP-43 and FUS/TLS are commonly involved in ALS through different pathways, their common implication in gene expression/RNA homeostasis suggests that alterations of expression regulation may represent key events in ALS pathogenesis [[15], [16], [17]], supporting also by the important involvement of numerous RBP in ALS.

In fact, there are various examples of RBP involved in ALS pathogenesis, such as hnRNPA2B1, MATR3, TAF15 and TIA1 [[18], [19], [20], [21], [22]].

Heterogenous Nuclear Ribonucleoprotein A2B1 (hnRNPA2B1) already associated with neurodegeneration [18]. Its function in the nervous system is unclear, but transcriptome-wide cross-linking and immunoprecipitation in mouse spinal cord discover UAGG motifs enriched within ∼2500 hnRNP A2/B1 binding sites and an unexpected role for hnRNP A2/B1 in alternative polyadenylation. HnRNP A2/B1 loss results in alternative splicing (AS), including skipping of an exon in ALS-associated d-amino acid oxidase (DAO) that reduces d-serine metabolism [19].

Also, mutations in Matrin 3 (MATR3) have been associated to ALS and its RNA binding domains are homologous to those found in the heterogeneous nuclear ribonucleoproteins (HNRNPs) [20]. MATR3 binds multiple nucleolar proteins, proteins associated with stress granules, and proteins involved in RNA splicing/processing. The proteins that have been most consistently reported to interact with MATR3 are proteins known to be involved in RNA processing or have been associated with RNA stress granules. In a recent study, it has been observed that the RNA recognition motif 2 (RRM2) of MATR3 is a major modulator of the intranuclear distribution of MATR3 with deletion of this motif causing the protein to exhibit an altered distribution into spherical structures. Interestingly, loss of the RRM2 domain induced a large number of apparently aberrant proteins [21].

TATA-Box Binding Protein Associated Factor 15 (TAF15) binds to the 3′UTR of select mRNAs, thus this implies a role in RNA stability [22]. Wild-type TAF15 is pathogenic when present at high levels, while ALS-mutant forms of TAF15 can enhance the pathogenic phenotypes of TAF15 over that of wild-type protein. Mutant forms may alter the normal activities of TAF15 by disrupting RNA or protein interactions by altering the subcellular localization of TAF15, and as a result prevent nuclear functions of TAF15, promote toxic cytoplasmic functions, or both. Indeed, ALS-mutant forms of TAF15, namely G391E, R408C, and G473E, had a higher propensity to localize to cytoplasmic foci [23].

Mutation in T-cell restricted intracellular antigen-1 gene (TIA1) was identified as a cause of amyotrophic lateral sclerosis (ALS) and frontotemporal dementia (FTD) [24]. TIA1 is an RNA binding protein that plays a central role in the formation of stress granules (SG) that form in response to environmental stress to temporarily store and protect mRNA [25]. Given that TDP-43 is recruited into SG under stress conditions [26] it has been showed that prolonged localization of TDP- 43 within persistent SG promotes TDP-43 aggregation and reduces its solubility. There is currently no evidence for any abnormal distribution or accumulation of TIA1 protein in postmortem brain tissue of TIA1 mutation carriers, but its mutation could be indirectly linked to TDP43 aggregation [27].

Also, given the deregulations associated to non coding RNAs (small and long) in ALS, these seem to have an implication in ALS disease, even if the available data are still few and do not describe the possible mechanisms in relation to the pathogenesis [6,7]. In this review, we have collected all the presented data about lncRNAs and ALS to offer an overview about this class of ncRNAs and their possible role in ALS disease.

## 2 Long non-coding RNA (lncRNAs)

Thanks to the Encyclopedia of DNA Elements (ENCODE, 2003) Project [28], which led to catalogue the functional elements encoded in human genome by using high-throughput methods, we know that 98% of the human genome is composed of non-coding sequences. Moreover, the inclusion of lncRNAs identification in the Whole Exome Sequence project has allowed the investigation of these RNAs, and part of them has been demonstrated to play a role in RNA expression regulation. Lots of studies have demonstrated that lncRNAs, transcripts of more than 200 nucleotides that are not translated into proteins, are significantly involved in different biological processes; thus they are especially important to the studies of human biology and diseases [29]. LncRNAs are currently divided into five groups named intergenic, intronic, bidirectional, sense and antisense, while the most cases are described generically as lncRNAs for the difficulty to organize this kind of RNA in specific classes.

### 2.1 LncRNA nuclear-enriched abundant transcript 1_2 (NEAT1_2T)

The complexity of ALS disease is due to the numerous different cellular features, one of those are the “paraspeckles”, nuclear bodies formed by a set of specialized proteins and RNAs, such as the lncRNA Nuclear-Enriched Abundant Transcript 1_2 (NEAT1_2T) [30], that was identified associated to ALS in brain [31]. This long non-coding RNA contains a GC-rich sequence and it has been demonstrated to be predominantly expressed in spinal motor neurons in an early phase of the ALS pathological process. Interactions of NEAT1_2 with ALS-associated RNA binding proteins were investigated and it has been shown that TDP-43 and FUS/TLS were enriched in paraspeckles and bound directly to the transcript [31]. The direct binding of TDP-43 or FUS/TLS to NEAT1_2 lncRNA was verified through iCLIP data targeting TDP-43, where NEAT1_2 lncRNA was one of the target RNAs bound predominantly by TDP-43 in human brain tissue with TDP-43 proteinopathy and cultured cells (HeLa and SHSY-5Y) [32]. Also, the in vivo crosslinking of RNA-protein (FUS/TLS) in mouse brain has demonstrated that there is a consistent part of the nonredundant mapped reads belonging to annotated long noncoding RNAs [33]. Both TDP-43 and FUS/TLS are paraspeckle proteins, which are thought to be required for normal paraspeckle formation through the direct interactions with NEAT1_2 lncRNA [30], even though a study seems not to address their formation to TDP-43 [34]. The frequency of paraspeckle formation is highly increased during the early phase of ALS pathological course thus, it has been concluded that NEAT1_2 could act a scaffold of RNA binding proteins in the nuclei of ALS motor neurons [30].

Interestingly, also FUS is an integral component of paraspeckles [35] that significantly contributes to their stability by both regulating NEAT1 steady-state levels and maintaining the structure of this nuclear body. Therefore, FUS mutations may impair paraspeckle formation needed for an adequate response to stress [36].

### 2.2 CCND1 associated ncRNA (ncRNA_CCND1_)

Single-stranded low-copy-number ncRNA transcripts have also been associated to FUS/TLS (Fused in sarcoma/translocated in liposarcoma). FUS is a multifunctional protein component of the heterogeneous nuclear ribonucleoprotein (hnRNP) complex and it is implicated in cellular processes that include regulation of gene expression, maintenance of genomic integrity and mRNA/microRNA processing [37]. Defects in this gene result in ALS type 6 [38]. It has been demonstrated that this RNA-binding protein serves as regulatory sensor of DNA damage signals by binding and inhibiting CREB-binding protein (CBP) and p300 histone acetyltransferase activities on its target gene, cyclin D1 (CCND1) in human cell lines, halting the cell cycle progression. Unrecognized local transcripts, generated upstream of the CCND1 promoter, described in HeLa cells, are likely to interfere with this process and to be ionizing-radiation-enhanced ncRNAs transcribed from multiple 5’ regulatory regions of CCND1 (ncRNACCND1). ncRNACCND1 binds to FUS/TLS through a consensus sequence GGUG [39]. C-terminus of FUS interacts with the GGUG oligonucleotide which enhances the binding of FUS/TLS to p300 and CBP, inhibiting them. Mutations of GGUG to CCUC caused impaired binding resulting in dissociation from p300 and CBP. Taken together, these findings suggest that an RNA-dependent allosteric modification of FUS/TLS relieves the inhibitory function of the C terminus of FUS/TLS. Also, data suggest that FUS/TLS acts as a repressor specific for CCND1, in fact, CCND1 is downregulated in response to DNA damage signals, that correlated with decreased histone acetylation. ncRNACCND1 has not been described yet in ALS, but it would be interesting to evaluate its possible role in this pathology given the strong correlation to FUS.

## 3 Antisense long noncoding RNAs (lncRNA-AS)

Natural antisense RNA transcripts are lncRNAs that are transcribed from the opposite strand (OS) of protein-coding genes or a sense strand-derived mRNA [40,41], and therefore, share sequence complementarity. They mediate their function through transcriptional and epigenetic regulation [42], thus they do not simply block splicing or translation of protein coding genes [43]. In fact, they can exploit their function at different stage of gene regulation: pre-transcriptionally, transcriptionally and post-transcriptionally.

There are well documented examples of antisense-induced chromatin modifications, suggesting that lncRNA-AS guided gene silencing could well have broader significance [44]. Sequence complementarity can establish complex configurations, such as RNA-DNA duplexes and triplexes. Rather than being involved in direct complementary interactions, RNA folds may create a DNA-binding pocket in a manner analogous to the DNA-binding domains of a protein transcription factor [45]. These direct RNA–DNA interactions could efficiently and selectively target RNA signals to genomic loci correlated, for example, with DNA methylation or with transcriptional repression. It has been demonstrated that lncRNA-AS can act in two ways based on whether the antisense RNA acts in *cis* or in *trans*. In *cis*, the antisense RNA interacts with a gene transcribed from the same DNA region, whereas in *trans*, the interaction is with genes located at distant loci or even at other chromosomes [46].

Another mechanism, regarding antisense transcript-mediated gene regulation, is based on the predicted nuclear sense-antisense RNA duplex formation, which results in the modulation of sense mRNA expression. Antisense RNA may bind to the sense RNA, masking the splice sites and thereby changing the balances between splice variants [47], or antisense transcripts can cause alternative polyadenylation and termination of transcription. Moreover, antisense RNAs can affect mRNA nuclear transport and retention. A duplex may also form between sense and antisense RNA in the cytoplasm, leading to the formation of RNA hairpins affecting sense mRNA stability or translation [42].

LncRNA-AS can also function as molecular decoys, with sponge-like properties, taking away proteins from a specific location or recruiting proteins to activate or inhibit processes involved at any level of gene regulation [44]. Antisense RNAs have been demonstrated to interact with proteins because they possess distinct protein-binding domains that combine molecules together, supporting the assembly of protein complexes that may function as transcription activators or repressors [48].

In a paper recently published by our group, a strong deregulation of lncRNAs has emerged in Peripheral Blood Mononuclear Cells (PBMCs) from sporadic ALS patients compared to healthy controls. Interestingly, the most represented gene biotype associated with the disease was lncRNA-AS [16]. Moreover, there are few examples of antisense genes likely to be involved in cellular impairment caused by ALS.

### 3.1 Ataxin 2 antisense transcript (ATXN2-AS)

Ataxin 2 (ATXN2) is a coding gene recently linked to ALS by the association between the length of ATXN2 repeat expansion and risk of ALS [49]. ATXN2 protein localizes to the endoplasmic reticulum and Golgi apparatus and is widely expressed. Its function concerns a number of cellular pathways, including translation, mRNA maturation, energy metabolism and endocytosis [[50], [51], [52]]. Recently, it has been demonstrated that ATXN2 can act also as a RNA-binding protein and that it is involved in ALS pathogenesis due to the RNA-dependent interaction between mutant ATXN2 protein and TDP-43 [53]. About ATXN2, the antisense transcript ATXN2-AS has been recently described, ATXN2 locus is bidirectionally transcribed in ALS tissues with ATXN2 expansion [54]. The antisense transcript ATXN2-AS with a CUG repeat expansion is neurotoxic, and therefore may contribute to ALS pathogenesis. In their work Li et al. detected ATXN2-AS expression from both normal and expanded alleles in 3 human ALS lymphoblastoid lines that have intermediate CAG expansions in ATXN2 (31–32 triplets). They confirmed by sequencing the expression of expanded ATXN2-AS in ALS lymphoblastoid cells and assessed its toxicity in neuronal-like SK-N-MC neuroblastoma cells [54]. It has been suggested that the CUG transcript toxicity is dependent on the structure formed by the repeats [55]. CUG repeats form hairpin structures, the stems of which act with sponge-like properties, mentioned above, and sequester RNA-binding proteins causing disrupt in RNA metabolism.

### 3.2 Chromosome 9 open reading frame 72 antisense transcript (C9ORF72-AS)

(GGG GCC)n hexanucleotide expansion within the first intron of the C9orf72 gene is the most common genetic cause of both ALS and Fronto-Temporal Dementia (FTD), causing up to 40% of familial ALS and 25% of familial FTD [3]. Health individuals typically carry 2–10 repeats, whilst patients hundreds to thousands [56]. So far, five described transcripts have been identified and also more than one antisense transcript that arise the same promoter region but the transcription proceed in the opposite direction [3,[56], [57], [58]]. The function of the C9orf72 lncRNA is still unknown but seems that the first exon of C9orf72 lncRNA have different binding sites for miRNAs. Cellular toxicity has been proposed to result either from a gain of function or from a loss of function of the C9orf72 gene [59]. More than one toxicity mechanism has been linked to C9orf72-AS, including toxicity derived from dipeptide proteins translated from the repeat RNA (DPRs) through repeat-associated non-ATG initiated translation (RANT) [60]. RNA toxicity is led by the hexanucleotide repeats that are transcribed in both sense and antisense directions, resulting in nuclear aggregates of sense and antisense RNA. The RNA mechanism of toxicity may be caused by sequestering RNA-binding proteins [11]. C9orf72 repeat RNA forms secondary structures such as hairpins and G-quadruplexes, leading to the formation of intranuclear RNA foci, which are generally assumed to become toxic by the sequestration of RNA-binding proteins [61]. However, other studies have demonstrated that the burden of sense and antisense nuclear RNA foci did not correlate with the region-specific neurodegeneration observed in patients post-mortem tissue [62].

Different works have demonstrated that the expression of hexanucleotide RNA is extremely toxic to adult Drosophila neurons, resulting in dramatically shortened lifespan [63]. Although, insertion of interspersed stop codons within the hexanucleotide sequence, resulting in “RNA-only” constructs, avoid toxicity, suggesting that it is primarily mediated by DPRs [63]. Despite these advances, the potential role of sense and antisense RNA toxicity in C9orf72 expanded-associated ALS is not fully understood. Several groups are trying to create antisense oligonucleotides (ASOs) targeting sense RNA stand [[64], [65], [66]] or G-quadruplex binding molecules, which target the sense strand specific G-quadruplex RNA secondary structure [[67], [68], [69]]. These approaches avoid the formation of sense RNA foci and the production of abundant sense dipeptide proteins. However, if the toxicity in patients is led by antisense RNA, this kind of intervention may be of limited clinical effectiveness.

A recent work studied the toxicity of sense and antisense C9orf72 RNA in Drosophila [70]. In order to study exclusively the RNA-mediated toxicity, they used RNA-only constructs to create a novel RNA-only Drosophila model to avoid the confounding effects of RANT DPRs. They generated Drosophila expressing ∼100 repeats sense and antisense RNA either as part of a processes polyadenylated transcript or intronic sequence and also flies expressing >1000 RNA-only repeats in the sense direction. The polyadenylated repeats RNA expressed in adult Drosophila neurons is largely cytoplasmatic in localization, while intronic repeats RNA forms intranuclear RNA foci, presumably because splicing of the intronic repeats prevents the nuclear export of repeat RNA. Despite these RNA foci are able to sequester endogenous Drosophila RNA-binding proteins; they found no evidence of toxicity arising due to sense and antisense repeat RNA. This model has provided evidence that in Drosophila neither cytoplasmic nor nuclear RNA are sufficient to induce a strong pathological phenotype [70]. The lack of neurotoxicity observed in RNA-only model is attributable to the lack of expression of DPRs. Indeed, the only toxic phenotype they observed was in flies with ∼100 repeat Sense-PolyA, where the expression of DPRs was higher. These results, therefore, imply that although repeat RNA is capable of sequestering Drosophila RNA-binding proteins, this is not sufficient to produce an overt phenotype associated with neurodegeneration in Drosophila. Sense and antisense RNA seem to be well tolerated in Drosophila, suggesting that dipeptide repeat proteins are a better target for therapeutic tools. An important role of RNA toxicity was recently characterized in a zebrafish model for C9orf72 ALS [71]. They injected RNA consisting of ∼70 GGGGCC repeats and antisense repeats CCCCGG in zebrafish embryos and compared motor axonal outgrowth to the control condition. RNA repeats dose dependently induced a motor axonopathy consisting of reduced axonal outgrowth and aberrant branching. These abnormalities were very similar to those described for mutant SOD1 and TDP43, two other genetic causes of ALS. The motor axonopathy appeared in absence of DPRs and also a synergistic role of low levels of DPRs was excluded. Therefore, the authors concluded that the presence of stretches of either sense or antisense C9orf72 hexanucleotide repeat RNA in the complete absence of any DPRs is sufficient to cause motor neuron toxicity in zebrafish model, supporting the existence of RNA toxicity.

## 4 LncRNAs profiling in ALS

Recently, different massive transcriptome profiles have been published in different tissues of ALS patients and matched controls showing and important deregulation in expressed genes [72,73] and in lncRNAs [16].

So, far, only one paper reported a transcriptome sequencing in peripheral blood mononuclear cells (PBMCs) from ALS patients [16]. PBMCs unveil different transcriptome profiles between unmutated sporadic ALS patients, mutated ALS patients (FUS, TDP-43 and SOD1) and healthy subjects concerning both coding and long non coding RNA [16]. A total of 293 differentially expressed lncRNAs was found in unmutated sporadic ALS patients, whereas a smaller amount of them was deregulated in mutated patients. The majority (184 transcripts) of deregulated long non coding transcripts of non-mutated ALS patients consists of antisense RNA, mostly unknown. Considering the most deregulated in non-mutated ALS group respect to healthy controls, it is evident that transcription pathway is highly involved, demonstrated the DNA binding and in transcriptional regulation as the most implicated one. In FUS mutated patients, 21 lncRNAs were identified, 11 of them are Antisense. For TDP-43 mutated patients 7 lncRNA-AS were detected, and only one was already described, SNAP25-AS. In ALS mice model, human TDP-43 decrease the RNA levels of synaptic proteins, such as SNAP25, so a role of SNAP25-AS in this deregulation cannot be excluded [74]. Finally in SOD1 mutated patients 2 novel antisense RNAs have been reported, one of these annotated as CKMT2 antisense. Transgenic mice overexpressing a mutant form of human superoxide dismutase showed a reduced creatinine kinase activity by inactivation of important target enzymes, including MtCK. Oxidative damage to the CK system in ALS may contribute to impaired energy metabolism and neurodegeneration [75].

## 5 lncRNAs in ALS animal model

The long non-coding transcriptome of mouse motor neurons (MNs) has been identified and characterized by taking advantage of an *in vitro* differentiation system generating MNs from embryonic stem cells. Moreover, by using mutant mouse MNs carrying ALS-associated FUS alleles (P517L), specific lncRNAs affected by this mutation have been pointed out and comparative analysis with human MNs derived *in vitro* from induced pluripotent stem cells have indicated that candidate lncRNAs are conserved between mouse and human [76]. A subgroup of lncRNAs has shown activation of the expression in the late stages of differentiation, when post-mitotic MNs are specified, possibly indicating a role for these molecules not only in MN differentiation but also in their mature physiology. Three of the conserved lncRNAs identified (Lhx1os, lncMN-1 and lncMN-2) were affected by FUS/TLS through a loss-of-function mechanism. In the case of Lhx1os and lncMN-1, the Protein Coding Genes (PCGs) expressed in divergent orientation also showed the same trend of deregulation, indicating a co-regulated response to FUS/TLS. Interestingly, these PCGs divergent from lncRNA genes were enriched for transcription factors involved in pattern specification [77], such as the one of laterally positioned lateral motor column MNs which are the MNs projecting to the dorsal muscles of the limbs [78] and were involved in controlling the expression of adhesion molecules, therefore participating in the establishment of axon trajectory [79]. In according to human data in PBMCs [16], specific lncRNAs have been described in FUS mutated patients and the main data concern PAXBP-AS1, antisense of a PAXBP that codifies for a protein fundamental for skeletal muscle development, suggesting a link between development and FUS mutations in ALS [80].

## 6 Concluding remarks

In this review we have reported the published data about the lncRNAs described associated to ALS even if the vision is still confused. What about the lncRNAs regulation on ALS linked genes? Up-regulation of SOD1 mRNAs in ALS patients, that the is a still unknown, may be related to any lncRNAs effect? FUS mutation may be linked to defects in development by lncRNAs expression?

As we know, ALS disease is still without any cure, in particular seems to be very important a medical approach at the first stage of the disease. A pharmacological approach on RNA that are deregulated at the early phase of ALS, such asNEAT1_2T, may be an interesting target. Moreover, the next step about lncRNAs is to understand wicth are the targets of their regulation, starting from the most deregulated lncRNAs founded in ALS patients by, for example, RNA pull-down experiments. This allows us to understand the gene expression regulation to hypothesize lncRNAs as a potential value in anti-ALS therapy. Considering that lncRNAs can be both positive and negative regulators of the transcription, they could be targeted using different approaches. RNAi mediated gene silencing provides a direct approach to selectively inhibit target molecules by means different agents like siRNA (small interfering RNAs), shRNAs (short hairpin RNAs), and miRNAs. AntagoNATs, ASOs that target antisense lncRNA, could be employed to up-regulate specific mRNAs/proteins by silencing the corresponding antisense lncRNA. Moreover, we could also block the molecular interactions of lncRNAs with their interacting protein partners by small molecule inhibitors that mask the binding sites on their interactors.

Finally, the available data may also open to use lncRNAs as biomarkers, future studies on asymptomatic patients will be fundamental to identify possible deregulated lncRNAs before the onset of symptoms for a preventive future treatment.

The published data has brought the light on the importance of lncRNAs and mRNAs regulation, but, on the other hand, is evident how still much to do to identify and understand the lncRNAs role in ALS that may offer numerous starting points for new investigations about pathogenic mechanism involved in ALS disease.

## Declarations of interest

None.
